# The Antitumor Effect of Heparin is not Mediated by Direct NK Cell Activation

**DOI:** 10.3390/jcm9082666

**Published:** 2020-08-18

**Authors:** Gustavo R. Rossi, Jenifer P. Gonçalves, Timothy McCulloch, Rebecca B. Delconte, Robert J. Hennessy, Nicholas D. Huntington, Edvaldo S. Trindade, Fernando Souza-Fonseca-Guimaraes

**Affiliations:** 1 Cellular Biology Department, Federal University of Paraná, Curitiba, Paraná CEP 81351-980, Brazil; goncalves.je49@gmail.com; 2 The University of Queensland Diamantina Institute, University of Queensland, Woolloongabba, QLD 4102, Australia; timothy.mcculloch@uq.edu.au; 3 Division of Molecular Immunology, The Walter and Eliza Hall Institute of Medical Research, Parkville, VIC 3052, Australia; DelcontR@mskcc.org (R.B.D.); hennessy.r@wehi.edu.au (R.J.H.); nicholas.huntington@monash.edu (N.D.H.); 4 Department of Medical Biology, Faculty of Medicine, Dentistry and Health Sciences, University of Melbourne, Parkville, VIC 3010, Australia; 5 Immunology Program, Memorial Sloan Kettering Cancer Center, New York, NY 10065, USA; 6 Department of Biochemistry and Molecular Biology, Biomedicine Discovery Institute, Monash University, Clayton, VIC 3800, Australia

**Keywords:** NK cells, cancer, heparin, antitumor responses

## Abstract

Natural killer (NK) cells are innate lymphocytes responsible for the elimination of infected or transformed cells. The activation or inhibition of NK cells is determined by the balance of target cell ligand recognition by stimulatory and inhibitory receptors on their surface. Previous reports have suggested that the glycosaminoglycan heparin is a ligand for the natural cytotoxicity receptors NKp30, NKp44 (human), and NKp46 (both human and mouse). However, the effects of heparin on NK cell homeostasis and function remain unclear. Here, we show that heparin does not enhance NK cell proliferation or killing through NK cell activation. Alternatively, in mice models, heparin promoted NK cell survival in vitro and controlled B16-F10 melanoma metastasis development in vivo. In human NK cells, heparin promisingly increased interferon (IFN)-γ production in synergy with IL-12, although the mechanism remains elusive. Our data showed that heparin is not able to increase NK cell cytotoxicity.

## 1. Introduction

Immune checkpoint inhibitors have revolutionized cancer therapy by reactivating tumor-resident cytotoxic lymphocytes. Checkpoint inhibitors primarily block inhibitory pathways in tumor-resident T cells; however, interest in other effector populations, such as natural killer (NK) cells, is growing. NK cells possess an innate ability to detect cellular transformation and are key to cancer immunosurveillance, particularly in settings of metastasis (e.g., melanoma) or hematological cancers [1]. Therapeutic strategies for metastatic melanoma have been changing over time to achieve optimal outcomes, and as such, chemotherapy has been increasingly replaced with more targeted immunotherapies [2]. Current immunotherapy regimens for treating melanoma are based on high doses of proinflammatory cytokines (IL-2 or Interferon-α) [3] or monoclonal antibodies to block antitumor immune checkpoints and stimulate cytotoxic T lymphocytes (anti-CTLA-4L and anti-PD-1L) [4,5,6]. NK cells are emerging as targets for cancer immunotherapy due to many advantages. For instance, they present reduced risk of autoimmune disease compared to cytotoxic T lymphocytes [1]. Additionally, as innate lymphocytes that are poised to kill transformed or infected cells [7], NK cells do not require prior clonal selection and expansion by specific antigens [1]. Instead, NK cells have many stimulatory (e.g., NKG2D, NKp30, NKp44, NKp46, CD16) and inhibitory (e.g., PD-1, TIM3, TIGIT, KIR/Ly49, NKG2A) surface receptors that directly define their activation and cytotoxic state [8,9]. At the same time, cancer cells create a complex tumor microenvironment to evade immunosurveillance by NK cells [10], such as using the TGF-β superfamily to impair NK cell antitumor responses by suppressing their metabolism, killing capacity, and inducing tissue residency characteristics [11,12,13]. The discovery of new immune stimulators could overcome this immune suppression of NK cells by tumors. 

Advanced-stage metastatic melanoma patients are at high risk of developing the clinical condition of cancer-associated thrombosis (CAT) [14], a major adverse effect that can often result in death of the patient [15]. The process starts with tumor cells ability to activate the coagulation cascade, leading to activation/production of Xa and VIIa factors, thrombin, and fibrin, resulting in platelet recruitment and culminating in thrombus formation [15,16]. The classic anticoagulant drug heparin [17] is used in oncology clinics to avoid thrombus in the bloodstream [18], where heparin-treated patients show increased survival, usually associated with a lower incidence of CAT [19]. However, the possible direct antitumor effects of heparin are multifactorial. Heparin may act by reducing the activity of extracellular matrix remodeling enzymes, such as heparanases and metalloproteases [20]. Aside from the inhibition of many coagulation cascade components, heparin binds to P-selectin on the surface of platelets, blocking their adhesion to tumor cells and making them more visible to the immune system [21]. The combination of such factors leads to reduction of metastasis in a number of animal models, such as colon and mammary cancer, melanoma, and adenocarcinoma [22,23,24,25]. Some reports suggest a potential interaction between heparin and NK cell stimulatory receptors, such as NKp30, NKp44 (human) and NKp46 (both human and mouse) [26,27,28]. However, whether these interactions can indeed lead to activation of NK cell antitumor responses is still unanswered. Herein, we investigated the hypothesis that heparin could activate NK cell antitumor responses by increasing their cytotoxic capacity against tumor cells. 

## 2. Experimental Section

### 2.1. Ethics

Animal experiments followed the National Health and Medical Research Council (NHMRC) Code of Practice for the Care and Use of Animals for Scientific Purposes guidelines and were approved by the Animal Ethics Committees at both the Walter and Eliza Hall Institute (WEHI) and the University of Queensland Diamantina Institute (UQDI). All procedures performed in studies involving human participants were in accordance with the ethical standards of the Human Research Ethics Committee at WEHI and UQDI. 

### 2.2. Heparin

Unfractionated heparin was purchased from BioIberia (Bacelona, Spain). For in vivo assays, heparin was dissolved in PBS (Gibco—Thermo Fisher Scientific, Waltham, MA, USA) (2.5 mg/mL), and for in vitro assays, heparin was dissolved in RPMI medium (Gibco) (5 mg/mL). Both solutions were sterilized by filtration using 0.22-µm syringe filters.

### 2.3. Mice

C57BL/6 (WT), *Rag2^−/−^γc^−/−^* (immunocompromised), or *Ncr1^−/−^* (NKp46-deficient) mice were bred and maintained under specific pathogen-free conditions at WEHI or UQDI. All animal experiments were performed using an age range of 8–12 weeks.

### 2.4. Cell Lines

B16-F10 murine melanoma and A375 human melanoma cells were maintained at 37 °C and 5% CO_2_ in DMEM (Gibco) supplemented with 10% fetal bovine serum (FBS) (Gibco), 1% GlutaMAX (Gibco), 1% non-essential amino acids (NEAA) (Gibco), 10 mM HEPES (Gibco), and 1% penicillin/streptomycin (Gibco). YAC-1 murine leukemia cells were cultured in RPMI-1640 supplemented with 10% FBS, 1% GlutaMAX, 10 mM HEPES, 1% NEAA, and 1% penicillin/streptomycin.

### 2.5. NK Cell Isolation and Culture 

NK cells from spleens of WT and *Ncr1*^−/−^ mice were isolated by organ maceration, followed by selection using a mouse NK cell isolation kit (Miltenyi Biotec, Bergisch Gladbach, Germany). For human NK cell isolation, peripheral blood mononuclear cells (PBMCs) were first isolated from fresh umbilical cord blood by Ficoll-Paque density (1.077 g/mL) centrifugation (Sigma-Aldrich, St. Louis, MO, USA). NK cells were enriched from resulting PBMCs by following negative selection using the EasySep Human NK Cell Isolation Kit (Stem Cell Technologies, Vancouver, BC, Canada). Mouse and human NK cells were maintained in RPMI 1640 media supplemented with 10% FBS, 1% sodium pyruvate, 1% Glutamax, 1% NEAA, 10 mM HEPES, 0.1% 2-mercaptoethanol (Gibco), 1% penicillin/streptomycin, and human rIL-15 (Peprotech, Cranbury, NJ, USA).

### 2.6. Tumor Model

B16-F10 cells (2 × 10^5^) were injected intravenously via the lateral tail vein into C57BL/6 mice or (1 × 10^5^) into *Rag2^−/−^γc^−/−^* recipients reconstituted with NK cells as previously described [29]. Briefly, fresh isolated NK cells from WT or *Ncr1^−/−^* (4 × 10^5^) were injected via the lateral tail vein into *Rag2^−/−^γc^−/−^* mice, 12 h after tumor cell inoculation.

After 24 h of tumor cells injection, animals were treated with 10 mg/kg of heparin, with subcutaneous (sc) injections of 100 μL heparin solution (2.5 mg/mL) or PBS (control) every second day for 14 days. At the endpoint, lungs were harvested, washed twice in PBS, and fixed in Fekete’s solution overnight [30] to macroscopically count surface metastases.

### 2.7. NK Cell Proliferation Assay

Fresh isolated mouse NK cells were incubated with Cell Trace Violet (CTV) (Thermo Fisher Scientific) according to the manufacturer’s instructions, and 1 × 10^4^ labeled cells were seeded into 96-well V-bottom plates (Corning Inc., Corning, NY, USA) in culture media (200 µL/well) supplemented with rIL-15 (50 ng/mL), with or without heparin (100 µg/mL). Time endpoints (0, 24, 48, 72, 96, and 120 h) were assessed on a BD FACS Verse cytometer (BD Biosciences, San Jose, CA, USA). Flow cytometry analysis was performed using FlowJo X (BD Bioscience) software, and division numbers were determined using the precursor cohort-based method [12,31]. 

### 2.8. IFN-γ Production

Human NK cells (1 × 10^4^) were seeded into 96-well V-bottom plates in culture media (200 µL/well), containing rIL-15 (50 ng/mL) and rIL-18 (Miltenyi Biotec) (50 ng/mL), with or without heparin (1 or 100 µg/mL), and incubated for 24 h. IFN-γ quantification was performed on the culture supernatant using the ELISA human IFN-γ Kit (R&D Systems, Minneapolis, MN, USA), and compared to an analytical standard curve. As a positive control for IFN-γ production, a group was incubated with rIL-12 (Miltenyi Biotec) (10 pg/mL). Absorbance was measured using a VICTOR3 plate reader (PerkinElmer, Waltham, MA, USA).

### 2.9. Mouse Target: Effector Cell Co-Cultures

Target tumor cells (B16-F10 or YAC-1) were labeled with 15 µg/mL Calcein AM (Thermo Fisher Scientific) for 30 min at 37 °C, as previously described [32]. Murine NK cells were cultured for 24 h in media containing rIL-15 (20 ng/mL), with or without heparin (10 or 100 µg/mL), and then used to perform a 4 h co-culture assay with B16-F10 or YAC-1 labeled cells in different ratios in a 96-well round bottom microplate. The plate was then centrifuged (300× *g* for 5 min) and the supernatant was collected and transferred to opaque 96-well plates (PerkinElmer). Fluorescence emission was measured with a CLARIOstar microplate reader (BMG Labtech, Ortenberg, Germany).

### 2.10. Human Target: Effector Cell Co-Cultures

A375 melanoma cells were detached with TrypLE (Gibco) and co-cultured for 4 h with human NK cells previously stained with CTV and cultured for 24 h in media containing rIL-15 (50 ng/mL), with or without heparin (1 or 100 µg/mL), in a 4:1 ratio into a 96-well V-bottom microplate. Cells were stained with Fluorescein isothiocyanate (FITC)-conjugated Annexin V and propidium iodide (PI) (BD Biosciences) according to manufacturer’s instructions, and dying or dead cells were assessed on a BD FACS Verse cytometer. Flow cytometry analysis was performed using FlowJo software.

### 2.11. Statistical Analysis

Statistical analyses (as indicated in the Figure legends) were performed using GraphPad Prism 8 software (GraphPad Software, San Diego, CA, USA).

## 3. Results

### 3.1. Heparin Does Not Depend on NK Cells Activation to Reduce B16-F10 Lung Metastasis

**Figure 1:**
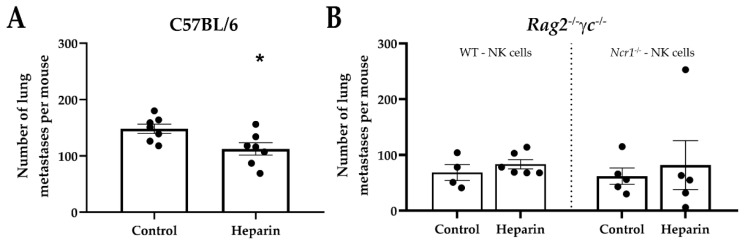
Heparin reduced the number of melanoma lung metastases. (**A**) C57BL/6 mice were injected intravenously with 2 × 10^5^ B16-F10 melanoma cells and treated with heparin (10 mg/kg every 2 days, subcutaneously), starting 1 day after tumor inoculation. After 15 days, mice were euthanized, and lung metastases were macroscopically counted. Graph is representative of two independent experiments. (**B**) *Rag2^−/−^γc^−/−^* recipients were injected intravenously with 1 × 10^5^ B16-F10, inoculated with 4 × 10^5^ sorted WT or *Ncr1^−/−^*(NKp46-deficient NK cells) 12 h later, and treated with heparin as in A. An unpaired *t*-test was used to compare differences between groups, where * *p* < 0.05 was used to compare to control.

To assess whether a heparin administration and treatment schedule could affect B16-F10 lung metastasis formation as previously suggested [33], C57BL/6 mice were intravenously injected with B16-F10 cells and treated with heparin (10 mg/kg) every second day for 14 days. Heparin-treated mice showed a 25% reduction in the number of visible metastases (Figure 1A). In vivo control of B16-F10-induced lung metastasis is largely NK cell dependent [34,35]. Therefore, we hypothesized that the metastases reduction in heparin-treated mice was due to NK cells activation since in silico analysis has predicted NKp46 as a potential receptor for heparin on NK cells [26,28]. To investigate that, we injected *Rag2^−/−^γc^−/−^* mice with B16-F10 cells, and reconstituted the mice with WT or NKp46-deficient (Figure 1B) NK cells after 12 h, followed by the same heparin treatment schedule as above. However, we failed to observe any differences on lung metastases regarding heparin treatment at the endpoint, suggesting that the interaction of NKp46 with heparin was not the responsible factor for reducing metastasis in this model.

### 3.2. Heparin Does Not Increase In Vitro Proliferation of Murine NK Cells

**Figure 2:**
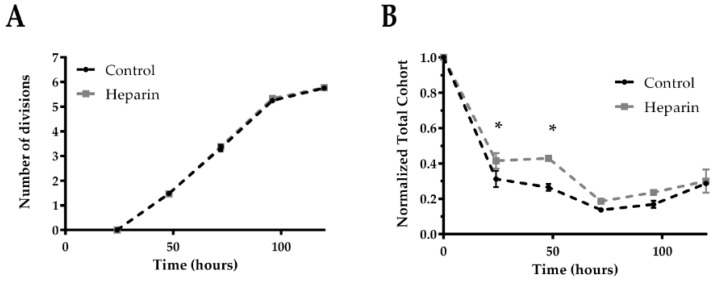
Heparin stimulates murine NK cells survival, but not proliferation in vitro. NK cells were stained with CellTrace violet (CTV) and plated in 96-well plates in the presence of 50 ng/mL rIL-15 and 100 µg/mL heparin and evaluated by flow cytometry every 24 h. The number of divisions (**A**), and total cohort (**B**) were analyzed. Data of three technical replicates of one representative independent experiment out of three. Data are presented as mean ± SEM. Two-way ANOVA was used to compare differences between groups, where * *p* < 0.05 was considered for statistical significance.

Murine splenic NK cells were exposed to heparin and assayed for cell proliferation kinetics as previously described [12,31]. Interestingly, while heparin does not appear to affect NK cell division rates (Figure 2A), using total cohort number analysis to estimate survival of NK cells [31], we observed that heparin was able to enhance the survival of NK cells in vitro (Figure 2B). Addition of heparin in cell cultures for enhanced manufacturing of NK cell products has previously been considered [36] as it is an effective replacement for stroma [37], and this might be because of the survival stimulus that we showed here. 

### 3.3. Heparin Does not Increase the Killing Capacity of Murine or Human NK Cells 

**Figure 3:**
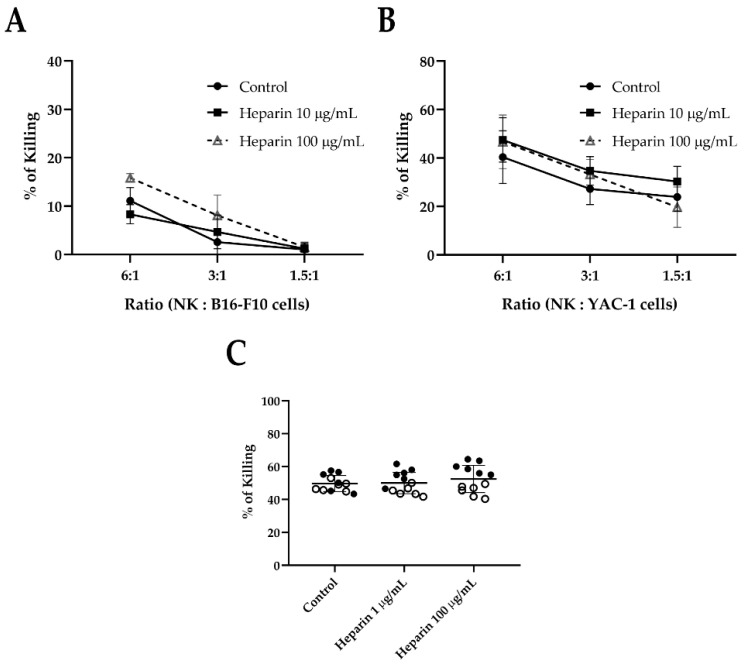
Heparin does not increase the killing capacity of NK cells. (**A**,**B**) NK cells isolated from C57BL/6 mice were cultured in the presence of 20 ng/mL of rIL-15 and heparin (10 or 100 µg/mL). After 24 h, NK cells were incubated for 4 h with previously Calcein AM-stained B16-F10 (**A**) or YAC-1 cells (**B**). Killing quantification was determined by the intensity of fluorescence in the supernatant and compared to control of each experiment. Each symbol in the scatterplots represents the average of three biological replicates (presented as mean ± SEM). Two-way ANOVA was used to compare differences between groups. (**C**) NK cells isolated from human peripheral blood mononuclear cells were cultured in the presence of rIL-15 (50 ng/mL), with or without heparin (1 or 100 µg/mL). After 24 h, NK cells were labeled with CTV and co-cultured with A375 cells (ratio 4:1—NK:A375 cell). After 4 h, cells were stained with Annexin V-Fluorescein isothiocyanate (FITC) and propidium iodide and evaluated by flow cytometry. Dead tumor cells were considered CTV^-^, Annexin V^+^, and/or PI^+^. Each point represents technical replicates from two independent experiments (represented by full and empty symbols; presented as mean ± SEM). An unpaired *t*-test was used to compare differences between groups, with *p* < 0.05.

To further investigate whether heparin could affect NK cell effector functions such as cytotoxicity, murine NK cells were pre-treated with heparin for 24 h, and then co-cultured with B16-F10 or YAC-1 cells. No difference in NK cell killing capacity was observed when treated with heparin across different NK:target cell ratios (Figure 3A,B), suggesting that heparin has no potential for stimulating NK cell cytotoxicity in this system. Human NK cells were also cultured in the presence of heparin, then co-cultured with A375 human melanoma cells, and again, no difference in NK cell killing capacity was observed (Figure 3C).

### 3.4. Heparin Increases IL-12-Mediated IFN-γ Production in Human NK Cells

**Figure 4:**
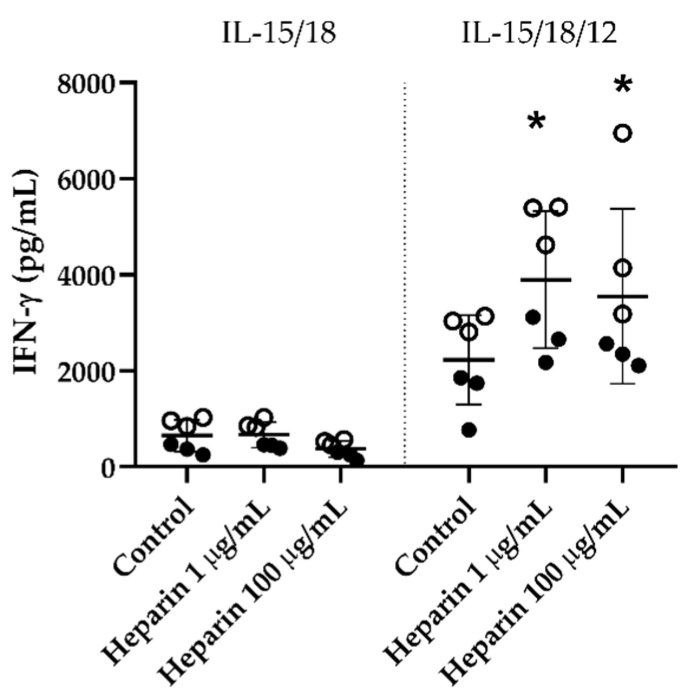
Heparin further increases IL-12-mediated IFN-γ production by human NK cells. NK cells isolated from umbilical cord blood were cultured in the presence of rIL-15 (50 ng/mL) and rIL-18 (50 ng/mL), with or without heparin (1 or 100 µg/mL) or rIL-12 (10 pg/mL) for 24 h. The supernatant was collected and IFN-γ quantified by ELISA. Each point represents technical replicates from two independent experiments (represented by full and empty symbols; presented as mean ± SEM). An unpaired *t*-test was used to compare differences between groups, where * *p* < 0.05 was used to compare to control.

We next evaluated the potential activation of NK cell effector functions by heparin. Human NK cells were cultured in the presence of heparin for 24 h and the production of IFN-γ was evaluated. Heparin did not increase the production of IFN-γ by NK cells cultured with rIL-15 and rIL-18. However, in the presence of the stimulatory cytokine IL-12, which drives production of IFN-γ and activation of NK cells [1], heparin significantly further enhanced IFN-γ production by NK cells (Figure 4) 

## 4. Discussion

Considering that the average cost for developing a new drug is now more than 1 billion US dollars [38], repurposing of “old” drugs to treat other diseases is an attractive proposal [39]. Heparin has been used in clinics since 1935, mainly due to its anticoagulant activity [17]. Considering that heparin may be a ligand for stimulatory receptors on NK cells (NKp30, NKp44 and NKp46), and it is currently used for NK cell product manufacturing purposes [36], in addition to other polysaccharides with potential to stimulate NK cells [40,41,42], we evaluated the capacity of heparin to stimulate and increase killing capacity of NK cells. 

Our results showed that heparin can reduce lung colonization by B16-F10 cells, which is in accordance with the literature for different types of cancer [33]. However, we failed to observe a direct association of heparin, NK cells, and the activating receptor NKp46, reinforcing previous suggestions that the antimetastatic effect is actually mediated by a direct effect of heparin over the B16-F10 cells (as previously suggested by others [43,44,45]), rather than a promotion of NK cell cytotoxicity. Corroborating our data, we also did not observe increased killing capacity of NK cells against both murine and human tumor cells in our experimental in vitro conditions. Therefore, heparin antimetastatic activity is likely mainly related to inhibition of coagulation, heparanase, and P-selectin interactions [46], avoiding cancer cell activation of the coagulation cascade to recruit a protective layer of platelets, and increase inflammation and angiogenesis [14]. 

A classical outcome of NK cell activation is the production of IFN-γ [47]. Our results showed that heparin alone could not stimulate IFN-γ production. However, when human NK cells were simultaneously cultured with the pro-inflammatory cytokine IL-12 and heparin, enhanced IFN-γ production was observed. IL-12 has a heparin binding site on the p40 subunit [48,49], and thus this interaction could potentially enhance IL-12 effect on NK cells, providing an explanation for our observed results. Similarly, another study using the human NK cell line NK-92MI also showed an increase of IFN-γ in the presence of heparin and IL-12 [50]. This increased IFN-γ production by NK cells could be related to the interaction of IL-12 with heparin rather than a direct effect of heparin on NK cells through receptors such as NKp46. 

Our results suggest that heparin is unlikely to be a direct stimulator of human or murine NK cell killing capacity. However, it is worth noting that the heparin binding sites in the stimulatory receptors (NKp30, NKp44, and NKp46) might have been already occupied by an endogenous ligand, such as heparan sulfate (which has a composition/structure very similar to heparin [51]) chains derived from the proteoglycan syndecan-4 [52]. If this is the case, then the externally added heparin cannot target its binding sites in order to activate the NK cells. 

Despite its lack of direct action on stimulatory NK cell receptors, the mechanism behind the heparin antimetastatic effect still needs to be further explored. Whether heparin interacts with other cell types that could exert beneficial off-target effects on NK cells is an exciting avenue to explore and has the potential to enhance combination therapies with direct NK cell-stimulating antibodies or cytokines.
